# Impact of Green Human Resource Management on Service Recovery: Mediating Role of Environmental Commitment and Moderation of Transformational Leadership

**DOI:** 10.3389/fpsyg.2021.710050

**Published:** 2021-10-21

**Authors:** Umer Iftikhar, Khansa Zaman, Mahmood Rehmani, Wajeeha Ghias, Tahir Islam

**Affiliations:** ^1^Department of Leadership and Management Studies, National Defence University, Islamabad, Pakistan; ^2^Faculty of Management Science, Shaheed Zulfikar Ali Bhutto Institute of Science and Technology University, Islamabad, Pakistan; ^3^Department of Business Administration, University of Sialkot, Sialkot, Pakistan; ^4^School of Economics and Management, Tongji University, Shanghai, China; ^5^Department of Management, Faculty of Management, Prague University of Economics and Business, Prague, Czechia

**Keywords:** employee environmental commitment, green service recovery, green HRM practices, transformational leadership, hotel industry

## Abstract

Greening of the hotel industry can be achieved through employees' green service recovery performance (GSRP) of employees to determine environment-friendly or unfriendly issues driven by environmental commitment and green human resource management (HRM) practices. This article attempts to resolve the research problem by analyzing the moderating effect of transformational leadership style on green HRM practices with employee environmental commitment (EEC) and GSRP in the hotel industry. The present study recruited 489 front-line employees (FLE) and their 24 direct managers. The research results reveal that environmental commitment of employee mediates the relationship between green HRM practices and GSRP. Moreover, the transformational leadership style moderates the relationship between green HRM practices and employee environmental commitment in the hotel industry. The study has critical insights and implications for hotel managers and theory.

## Introduction

At presently, sustainability toward the environment has gained much attention from stakeholders. As a result, organizations adopt various strategies to fill their corporate social responsibility (CSR) (Kim et al., 2017). Sustainability is considered the sustained competitive advantage in the modern world business environment. Thus, the hotel industry is also trying its best to contribute to sustainable development goals (SDGs). Scholars have found that the hotel industry was abruptly adopting the “green” efforts, including enlightening employees regarding such prospects (Umrani et al., 2020). Correspondingly, a good number of research have been conducted on greening the hospitality industry.

Contemporary research has emphasized environmental-related organizational resources and policies that help foster environment-friendly behavior among employees. Green organizational resources are those which help to reinforce and build environmentally-friendly values and behavior among employees. The concept of Green human resource management (HRM) is defined as the utilization of HRM strategies to energize the practical utilization of assets inside business interventions and advance the reason for environmentalism, which further lifts worker morale and fulfillment. Similarly, earlier it was defined as utilizing HRM practices, philosophies, and policies to encourage environmental-friendly usage of corporate possessions and address any untoward damage emerging from environmental sensitivities in the organizations.

This study will take a holistic approach toward green HRM practices and green service recovery performance (GSRP). The HRM practices, including recruitment, performance appraisal, and training, are likely to enhance the performance of front-line employees (FLEs) and moderate and mediate outcome variable GSRP. Researchers such as Babakus et al. (2003) and Van Vaerenbergh and Orsingher (2016) reported that green HRM practices might influence commitment of the employees toward service recovery performance. Taking the research lens from literature and furthering the research of Luu (2018) and Umrani et al. (2020), this study opted to analyze GSRP as an outcome from HRM practices mediated by the role of environmental commitment of employees through moderation of transformational leadership.

Green service recovery performance is built on the concept of service recovery performance (SRP). It is viewed as the perception of FLEs about their ability to resolve inimical environmental activities in a service organization to enhance positive behavioral outcomes (Babakus et al., 2003; Luu, 2018). Past literature researched and found that sustainability and environment-friendly efforts positively correlate with positive behavioral outcomes and hotel performance (Aşici and Bünül, 2012; Umrani et al., 2020). Literature on sustainability in the service sector has been extensively studied. To our best knowledge, limited research has been conducted on the concept of green recovery performance in service organizations to enhance business performance. The study examines the underlying mechanism through which the pro-environmental behavior of employees on GSRP in the hotel industry. Moreover, the study also examines the moderating and mediating role of leadership on the relationship between HRM practices and green recovery performance.

## Literature

Green HRM practices are considered a comprehensive measure to predict environmental management mechanisms (Tang et al., 2018; Umrani et al., 2020). Organizations and various sustainable agencies use green HRM practices to determine the level of organizational sustainability (Chaudhary, 2018). Extant literature found that environment friendly or green HRM practices positively influence financial performance and good corporate image. Researchers believe that green HRM practices are the tools that should be used to formulate training and recruitment designs to foster the green or environmentally friendly behavior in employees (Dumont et al., 2017). For instance, Cherian and Jacob (2012) focused on the need to effectively build pro-environmental knowledge, beliefs, awareness, and attitudes among employees. Similarly, scholars found that the practices of green HRM should build performance appraisals in a way that it recognizes the efforts of employees by rewarding them against their contribution to the environment to reinforce values related to pro-environment (Cherian and Jacob, 2012; Renwick et al., 2016). Furthermore, Luu (2018) and Babakus et al. (2003) found that green HRM practices like training, performance appraisal, and recruitment have a fundamental role in enhancing green recovery performance. Drawing on the definition of Luu (2018), green service recovery is perceived as the action and abilities of FLE to drive friendly environmental activities for customer satisfaction. We antedate in the current study that practices related to green human resources will play a pivotal role in enhancing the GSRP of employees.

This hypothesis is deduced from the recent work of Luu (2018) and Umrani et al. (2020), which analyzed that GSRP is an outcome construct of green HRM practices. For instance, Mihardjo et al. (2020) found a significant impact of HRM practices on service recovery performance. Similarly, businesses are concerned with stability and sustainable performance and focused on the factors responsible for the required outcomes (Birou et al., 2019). Literature on behavioral HRM directs that attributions related to HRM are conditional to employee-related outcomes of HRM (Nishii et al., 2008). Thus, we employed attribution theory to individual and own abilities to explain the interpretations of an individual. This theory provides theoretical support that individuals form attributions to understand their environment and to predict future events with their abilities from attribution. The systematic attributions of individuals affect their subsequent beliefs, cognitions, motivations, affects, and behavior (Weiner, 1985). The individual attitudinal responses to HRM policies can be related to the attribution concept utilized to comprehend the intent of management in the implementation of HRM (Nishii et al., 2008). Luu (2018) also argues that employees who acquire training on green parameters and receive rewards against their green contributions mark external attributions about greening their organizations.

Recently, green hospitality literature found that tourists invested more energy to find the right destination for their leisure time. As a result, the hotel industry is trying to provide the best competitive environment to their visitor and applying various best approaches to satisfying them. This caused a rise in intense competition among the hotel industry chains. In addition, the growing demands of sustainability from the tourists push hotel management to conduct their operations greener (Mittal and Dhar, 2016). The intention behind designing green HRM practices is to develop pro-environmental qualities and practices among representatives. Such values introduce individuals toward the significance and the prosperity of the typical habitat and how it should be seen and treated by people (Tang et al., 2018). In line with this, green conduct and green HRM should plan training (Dumont et al., 2017), employee empowerment, reward, and recognition programs (Renwick et al., 2016) that adequately construct natural mindfulness, attitudes, knowledge, and qualities among the employees. Past research suggests that green HRM practices play a significant role in achieving the required organizational goals and sustained competitive advantage (Paillé and Mejía-Morelos, 2014). Corporations have accepted the key significance of the employees and their efforts toward a sustainable economy. For example, several studies confirmed that green HRM practice adoption is the best suitable approach to enhance sustainable employee behavior (Perez et al., 2009).

**Figure 1:**
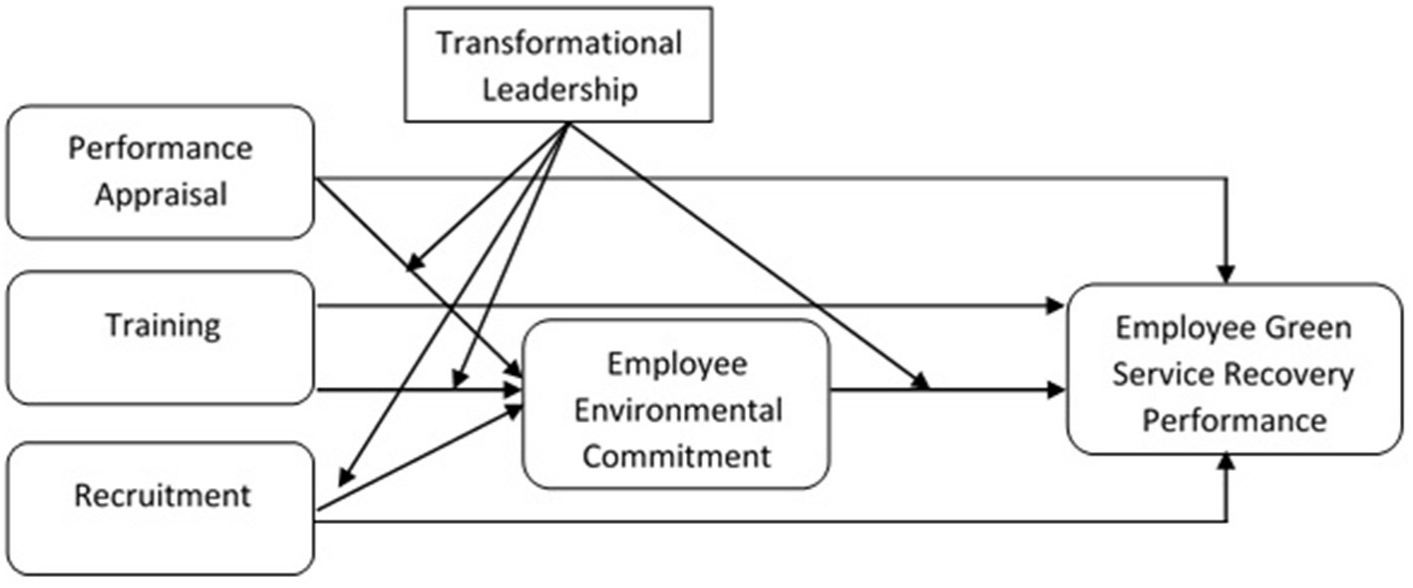
Conceptual framework by authors.

Green HRM practices ease the efforts of the organization to gain a sustained competitive advantage through training, workshops, and recruitment (Raineri and Paillé, 2016). Employees working in green-oriented associations have properly adhered to standards, norms, and operations to facilitate the firm to achieve its green goals (Pham et al., 2019). Thus, green HRM practices have a significant impact on the GSRP in the hotel sector. Therefore, we hypothesized as follows:

**H**_**1**_: Performance appraisal, training, and recruitment for pro-environmental behaviors significantly and positively affect the GSRP of employees (Figure 1).

### Moderating Role of Transformational Leadership

Extant literature claimed that envisioned green recovery performance, as outcomes of green HRM practices, will positively change the organization (Dumont et al., 2017; Luu, 2018). For example, Babakus et al. (2003) found that green recovery performance and green HRM practices determine organizational commitment to SDGs and sustainable performance. Based on the past findings, this study also hypothesized the influence of green HRM on GSRP (Fiske and Taylor, 1991). This hypothesis is supported by the attribution theory, which centers around people's underlying clarifications to interpret their personal and the mentalities and practices of others. Considering attribution theory, people use attributions to comprehend their condition and upgrade their capacity to figure future occasions. Also, through these attributions, people have efficiently conditioned their influences, inspirations, and practices (Weiner, 1985). Thus, the internal motivation of employees is the main antecedent to attain the GSRP. Green HRM practices may support worker ecological commitment utilizing transformational leadership. HRM frameworks are increasingly successful when they are lined up with the way of life of an association. Even though managers are commonly perceived as influential shapers of organizational cultures, little has been done to measure the role of leaders to add or oblige the adequacy of deliberately supported HRM frameworks (Schneider et al., 2017; Ren et al., 2020). Transformational leadership pass on ecological dreams for their organizations and fill in as impetus that lifts the relationship of green HRM practices and employee environmental commitment (Graves and Sarkis, 2018; Luu, 2018).

Furthermore, the previous examinations also suggested that a transformational leadership style should be utilized as a moderator in research models of green recovery performance (Luu, 2018). A leader who promotes and motivates employees toward environmental awareness for better ecological performance is the one who leads through green transformational leadership (Chen and Chang, 2013). This green administration persuades workers to hierarchical esteem objectives over close to personal goals and aids representatives in all circumstances, helps them whenever required, and imparts eagerness into workers to produce novel thoughts for the environment (Li et al., 2020). In this manner, a green transformational leader should manage and propel their workers to rehearse green practices to predict the green recovery performance of an individual (Zhou et al., 2018). In this manner, we conjecture that to certify the role of green human resource practices on the environmental commitment and GSRP of employees, a transformational leadership assumes a critical directing job to upgrade their relationship (Singh et al., 2020). Green HRM practices influence cognition and perception of employee through the impact of the green strategy of the organization. Notably, it helps build an environment of sustainability. These attributes will lead toward employee green recovery service through the lens of transformational leadership. The previous literature and research findings related to research on the impact of green HRM on green behavior outcomes framed this study to analyze the GSRP of employees as an outcome of green HRM practices. This will lead to the following hypotheses:

H_2_: Employee environmental commitment mediates the relationship between Green HRM practices and GSRP.

H_3_: Relationship of training, performance appraisal, and recruitment with employee environmental commitment is moderated by transformational leadership.

H_4_: Transformational leadership moderates the relationship between employee environmental commitment and the GSRP of employees (Figure 1).

## Methodology

This study collected data from the firms registered in the Security Exchange Commission of Pakistan (SECP) in 2017. We selected the hotels for the data collection with 75 employees and practicing a green strategy. It is difficult to get the correct data from the employees for the research in developing countries where firms do not believe in the research. We contacted HR managers and, in some cases, CEOs of hotel companies and presented them with the research problems that are only for good purposes. After soliciting their permission, the researchers of the present study promised to share the results of the current study with them. Among 244 hotels in Pakistan, 32 hotels met the above criteria. We contacted the HR managers and requested for the list of departments practicing green strategy and collected details and along with a list of department employees. We had telecommunication with the respective employees and sought their consent to participate in the survey. The survey was emailed to them upon their consent, and it was then emailed to all respondents, along with a cover letter. The cover letter explains the reason for the research along with the assurance for survey privacy. After 12 days, non-respondents were contacted through email reminders. The questionnaires had code numbers to match the responses of FLEs with their direct supervisors. The same code number was assigned to each FLE and their respective direct supervisor.

Data were collected in two wavelengths. Independent and dependant variables were separated to diminish common method bias, as Podsakoff et al. (2012) suggested. A time lag of 4 weeks was used based on the prior work of Luu (2017). Moreover, a minimum of two wavelengths of the data to test proposed meditation paths was adopted, as suggested by Cole and Maxwell (2003). Wavelength in the first T1, data related to performance appraisals, training, recruitment (green HR practices) and green HR practices, and employee environmental commitment was collected. After 1 month, the second wavelength (T2) started, and GSRP data were gathered from the FLEs and their supervisors. They have supervised them for a year, including the period of 2 wavelengths. We also collected controlled variables during the survey, which consisted of age, tenure, gender, and education. In the T1 survey, data were collected from 739 (67%) FLEs. In T2, 559 (51%) complete responses were accumulated using FLEs who were part of T1 by the crossing department with fewer than three employees (Addison et al., 2017). A final sample of 489 (44%) FLEs and 24 (75%) direct managers were garnered for final data analysis.

The average age of FLEs was 31.87 years SD (6.21), 32.81% of employees were female, and the organizational tenure of employees was 3.41 years (SD = 1.21). Out of the managers, eight were female with an average managerial term of 1.52 years (SD = 0.45) and 34 years (7.91). Chi-square tests were conducted to compare the first wavelength of data of employees with the second wave. The results confirm that the two groups of employees have no significant difference in terms of variables such as gender, age, tenure, and education.

### Measures

The items to be filled by FLEs were translated into the Urdu language (the national language of Pakistan). The scale to measure green HR practices was adapted from Agarwala (2003). The performance appraisal scale had six items, recruitment had six things (Agarwala, 2003), and training had six items. Transformational leadership was measured using the multifactor leadership questionnaire (MLQ). The items of green HR practices measured the pro-environmental behavior of employees. Cronbach alpha of performance appraisal, recruitment, and training was 0.78, 0.82, and 0.84, respectively. The GSRP (five items) with a Cronbach alpha value of 0.78 scale was adapted. Employee environmental commitment was gauged on a scale of 8 items (Raineri and Paillé, 2016) with an EEC of.83. The Likert scale (having anchors 1 = strongly disagree to 5 = strongly agree) was used to measure the responses.

The controlled variables included age (years), gender (1 = male, 2 = female), tenure at current hotel (years), and education (1 = Intermediate, 2 = Bachelors, 3 = Masters, 4 = diploma holders, and 5 = other). Tenure at the current hotel reflects the experience and knowledge of employees about the practices of their organizations. Education is an essential component to know the knowledge of an employee about pro-environmental behavior and the consequences of their actions on the environment.

## Results and Discussions

We employed a multilevel structure equation model (SEM), keeping in view the data type of multilevel responses from individuals within groups. Mplus 7.2 was utilized for hypotheses testing of multilevel SEM recently proposed by Preacher et al. (2010) to overcome the limitation of prevailing multilevel analysis used to predict mediating effects of the multilevel data. The data were screened for normality, multicollinearity, and outliers before main data analysis. Outlier scanning also included normality tests to sense outlier's presence and normality of data usually occur together. Proper treatment of the data was carried out, including transformation and deletion of variables (Hair et al., 2010). The observation with a skew range of 1 to 1.34 was retained. However, the observations with an alarming skew range of z = −4.65 and −4.91 were excluded from the main data analysis.

Missing data were analyzed through missing values analysis (MVA) in line with the prior research (Tabachnick and Fidell, 2014). The analysis indicated that the pattern of missing values was random, and the number of missing data did not exceed 4% in any of the variables. We used the imputation technique to treat missing data, including removing cases with missing data for further analysis (Hair et al., 2010).

Variance Inflation Factor (VIF) was employed to test the multicollinearity of the data, which indicated that all values are within a threshold value of 5 with the highest value of 3.87 (Hair et al., 2010). The values of VIF confirmed that multicollinearity is not an issue. Moreover, Cohen et al. (2003) proposed to lessen the imminent probability of multicollinearity while using moderation in analysis. Therefore, the independent continuous variables were centered by using mean, and then the values of means were multiplied to create a moderator term such as interaction term.

### Measurement Model

Multilevel factor analysis called for an elaborated test of multilevel data structure. The procedure was utilized for estimates of data structure as used in the research of Dyer et al. (2005). Table 1 of confirmatory factor analysis (CFA) reflected good fit indices between the hypothesized model of six factors and data. The model applied all fit indices. The fit indices exceeded the threshold values of 0.9 such as TLI = 0.97, CFI = 0.94, and IFI = 0.97 (Tabachnick and Fidell, 2014). The values of model fit, X^2/df^ = 319.41/162 = 1.97, maintained the minimum tolerable value of under 2 (Carmines and McIver, 1981). The values of error estimations were also in the required range with RMSEA = 0.045 and SRMR = 0.043.

**Table 1:** Comparison of measurement models.

**Model**	**X2**	**df**	**ΔX2**	**TLI**	**CFI**	**IFI**	**RMSEA**	**SRMR**
Hypothesized six-factor model	319.41	162		0.97	0.94	0.97	0.043	0.045
Four-factor model: Performance appraisal, training, and recruitment combined	354.91	174	49.21^**^	0.92	0.91	0.92	0.067	0.064
Three-factor model: Performance appraisal, training, recruitment, and transformational leadership combined	431.34	176	129.21^**^	0.94	0.93	0.94	0.047	0.049
Two-factor model: Performance appraisal, training, recruitment, transformational leadership, and GSRP combined	472.13	181	137.49^**^	0.89	0.88	0.90	0.141	0.135
One-factor model: All variables combined	634.56	190	335.81^**^	0.90	0.91	0.89	0.065	0.066

** *p < 0.01*.

The convergent and discriminant validity values were also tested and found in range with factor loadings crossing the benchmark value of 0.6, *t* > 2.01 for convergent validity and the square root of the average variance extracted (AVE) greater than its correlations for discriminant validity. In addition, for discriminant validity, we also contrasted the hypothesized six-factor model with an alternative model, which resulted in collapsing some of the factors for forming an alternative model. However, Table 1 indicated that hypothesized six-factor model is a good fit with construct distinctiveness against any other alternative model (Fornell and Larcker, 1981).

Moreover, the results of the multilevel CFA revealed that the factor structure, within-groups, and between-group level analysis, was strong in our hypothesized model. The model fit with-in groups (x2 = 342.34; df = 162; x2/df = 342.34/162 = 2.11; TLI = 0.95; CFI = 0.92; IFI = 0.91; RMSEA = 0.045; SRMR = 0.041) and between-groups (x2 = 302.98; df = 162; x2/df = 302.98/162 = 1.87; TLI = 0.91; CFI = 0.90 IFI = 0.94; RMSEA = 0.054; SRMR = 0.048) models.

Table 2 indicated the reliabilities of the measurement scales checked through AVE and CCR coefficients. Reliability values ranged from 0.71 (performance appraisal for pro-environmental behavior) to 0.84 (Recruitment of pro-environmental behaviorists), which were tolerable. The values of AVE also exceeded the cut-off value of 0.5 (Fornell and Larcker, 1981), ranged from 0.59 (EEC) to 0.69 (recruitment of pro-environmental behaviorists).

**Table 2:** Pearson's correlation, Composite Construct Reliability (CCR), and Average Variance Extracted (AVE).

	**Variables**	**Mean**	**SD**	**1**	**2**	**3**	**4**	**5**	**6**	**7**	**8**	**9**	**10**	**CCR**	**AVE**
1	Age	31.87	6.21	–											
2	Gender	1.72	0.75	0.02	–										
3	Education	2.11	0.87	0.01	0.03	–									
4	Tenure	3.41	1.21	0.02	0.07		–								
5	Performance appraisal for pro-environmental behavior	3.16	0.44	0.03	0.04	0.07	0.02	(0.78)						0.71	0.61
6	Training for pro-environmental behavior	3.65	0.68	0.05	0.01	0.04	0.01	0.21^*^	(0.81)					0.81	0.65
7	Recruitment of pro-environmental behaviorists	3.47	0.54	0.02	0.06	0.05	0.02	0.12^*^	0.31^*^	(0.83)				0.84	0.69
8	EEC	3.25	0.48	0.03	0.05	0.02	0.01	0.23^**^	0.33^*^	0.34^*^	(0.77)			0.76	0.59
9	Transformational leadership style	3.85	0.72	0.05	0.09	0.04	0.04	0.15^*^	0.29^*^	0.29^*^	0.31^*^	(0.8)		0.79	0.64
10	GSRP	3.33	0.47	0.08	0.02	0.05	0.09	0.27^*^	0.26^**^	0.43^*^	0.32^**^	32^*^	(0.81)	0.71	0.66

*SD, Standard Deviation; AVE, Average Variance Extracted; CCR, Composite Construct Reliability*.

*Parentheses reflect the square root of AVE. Pearson's correlation results reported*.

*p < 0.10*,

* *0.05*,

** *0.01*.

#### Common Method Variance

To treat the issue of CMV, a marker variable model was utilized (Lindell and Whitney). A marker variable of attitude toward buying luxury products was added into the survey, which was theoretically different from the study variables. The variable of the attitude of the marker was employed using a five-point Likert scale ranging from 1 = strongly disagree to 5 = strongly agree. The sample items were “I would love to buy a luxury car; I like to buy luxury brands.” The results donated a low CMV issue in the entire dataset as all significant zero-order correlations were significant after excluding the marker variable. Moreover, the interaction effects of four variables could be deflated by CMV (Siemsen et al., 2010).

#### Aggregation

Intra-class correlations coefficient (ICC) such as ICC (1) and ICC (2) were calculated to gauge the relevance of aggregating individual scores (transformational leadership style and green HR practices) to the scores of group level. ICC (1) indicates the proportion of variance in a variable caused by group membership, whereas ICC (2) represents the reliability values of group mean scores. The ICCs (1) for performance appraisal, training, and recruitment were 0.16, 0.13, and 0.14, respectively. The ICCs (2) were.74, 0.79, and 0.85, respectively. The ICC (1) for transformational leadership style was 0.19 and ICC (2) was 0.84. Besides, one-way ANOVA was utilized to determine the significant differences between groups regarding green HR practices and transformational leadership style. The result indicated significant F values with *p* < 0.01 for performance appraisal (F162, 1339), training (F162, 1336), recruitment (F162, 1329), and transformational leadership style (F162, 1321).

#### Descriptive Statistics

Table 2 demonstrates the values of means, SDs, Pearson's correlation, square root of AVE, CCR, and AVE of all study variables. Green HR practices such as performance appraisal, training, and recruitment displayed significant positive relationships with EEC (*r* = 0.76). Moreover, EEC had a significant positive association with the GSRP of employees (*r* = 0.71).

#### Hypotheses Testing

The present research indicates strong evidence for mediation hypotheses. The bootstrapping indirect effects and path analysis confirm role of EEC in fostering green recovery performance as an outcome of green human resource practices. The 95%CI represents a significant indirect effect by ruling out zero. The performance appraisal, training, and recruitment for pro-environmental behaviors significantly positively affect GSRP of employees as postulated in H1.

The postulated research hypotheses have significant positive coefficients such as β = 0.21 *p* ≤ 0.5, β = 0.29, *p* ≤ 0.5, and β = 0.34, *p* ≤ 0.1, respectively. Besides, EEC reflected on mediating the relationship between green HRM practices and the GSRP of employees. Performance appraisal posited a positive role in the shape of significant results with EEC (β = 0.24, *p* < 0.01), which indicated a significant positive correlation with the green recovery performance of employees (β = 0.37, *p* < 0.01). The indirect effect of performance appraisal on the GSRP of employees through EEC (effect = 0.07, CI [0.06, 0.15], *p* < 0.05) was found through bootstrapping. Similarly, training was a significant predictor of EEC (β = 0.41, *p* < 0.001). The EEC has indirect impact and was effect = 0.19, CI [0.11, 0.29], *p* < 0.01, using bootstrapping on employee training through GSRP. Recruitment also postulated a significant influence on EEC (*b* = 0.24, *p* < 0.01). The indirect bootstrapping effect indicated that recruitment has a significant indirect effect on the GSRP of the employee through the mediator, EEC (effect = 0.09, CI [0.04, 0.16], *p* < 0.05). All bootstrapping and path results corroborate the hypotheses of mediation ranges from H2.

To test the moderation effects of transformational leadership style in the relationship between green HRM practices and GSRP, the interaction terms were employed to test hypotheses 3. The results indicated a significant and positive interaction term (*b* = 0.2, *p* < 0.05) of predictor “performance appraisal” of green HRM practices X transformational leadership style. The interaction pattern was also evaluated at high and low mean values (one SD above and the low mean for high and low values, respectively) of transformational leadership style between performance appraisal and EEC. The interaction pattern reflected that performance appraisal strengthened EEC when transformational leadership was at a high slope value of 0.66 than the low slope of 0.11 (*p* < 0.05).

The moderation results of the second predictor indicated a significant and positive relationship (b = 0.25, *p* < 0.01) between training and transformational leadership style. The interaction pattern represented that training enhanced EEC when transformational leadership was at a high slope value of 0.77 than at a low slope value of 0.19 (*p* < 0.05).

Similarly, a significant and positive interaction term (*b* = 0.24, *p* < 0.05) was reflected between recruiting pro-environmental behaviorists X transformational leadership style. The interaction pattern showed that recruiting pro-environmental behaviorists increased EEC when transformational leadership was at a high slope value of 0.79 than at a low slope value of 0.16 (*p* < 0.05). Hence, hypotheses of interactive effects H3 were supported which represented that transformational leadership enhances the effects between green HR practices and EEC, which were further confirmed by respective slope tests.

Moreover, the results of moderation represented a significant and positive interaction term (*b* = 0.24, *p* < 0.05) of predictor (EEC) and moderator (transformational leadership style). The interaction pattern unveiled that EEC enhanced the green recovery performance of employees when transformational leadership was at a high slope value of 0.54 than at a low slope value of 0.07 (*p* < 0.05).

## Discussion

Past literature has examined and measured the role of green HRM practices on the GSRP. The present research contributes to the theory and practice. First, this study finding contributes to the HRM and leadership literature. This study results have added value to the green HR practices-GSRP relating it with the role of employee environmental commitment as a device to foster change in the perception of employees formed through the positive part of green practices elucidating in their GSRP. Moreover, this study examines the underlying mechanism (mediating-moderating) through which green HRM influences the GSRP. These moderating results show that transformational leadership strengthens the relationship between HRM practices and GSRP. This study extends the knowledge of transformational leadership in the hotel sector that green HRM and leadership support improve the GSRP. These findings align with extant literature, which acknowledges the role of employee commitment and leadership in achieving the desired goals. In addition, this research found that organization's initiatives to introduce green HRM establish a ground that includes employee environmental commitment and acquiescing the drive and strive of employees to develop environment-friendly activities in hotel services. This sustainable view will add value and be strengthened only if we engage leaders of the organization who transmit signals of environment-friendly behavior (i.e., transformational leadership) are engaged, and dependence on this path may contribute to the choices of employees of staying devoted to ecologically sustainable goals and to participate in green service recovery behavior. Future researchers may consider studying this model in different cultures. The role of other forms of leadership such as servant leadership and ethical leadership may also be focused on green HRM.

## Summary

Sustainability and organizational sensitivity to the environment have gained the attention of researchers and managers in recent years following the pressures from stakeholders on organizations for adoption and promotion of environmentally responsible behaviors. This global awakening is compelling organizations to become environmentally responsible and the hotel industry has no exception. Accordingly, hotels are also undertaking good-sized “green” efforts including enlightening employees regarding such prospects. Correspondingly, a good number of researches have been conducted on the greening of the hospitality industry.

Green service recovery performance (GSRP) is built on the concept of service recovery performance (SRP) which is viewed as the perception of front-line employees (FLEs) about their abilities to resolve inimical environmental activities in a service organization for enhanced customer satisfaction. The literature on sustainability and environment-friendly efforts in the hotel industry has drawn the attention of researchers and practitioners at a strategic level to ensure organizational sustainability for long period. Nevertheless, insufficient consideration is given to the concept of green recovery performance in service organizations to enhance business performance. This study seeks to resolve the research disparity on green service recovery performance in the hotel industry and deciphers part of the dusky check box following FLEs' green service recovery performance such as employees' pro-environmental behavior and the role of leadership as suggested by recent researchers for future studies.

## Data Availability Statement

The raw data supporting the conclusions of this article will be made available by the authors, without undue reservation.

## Ethics Statement

Ethical review and approval was not required for the study on human participants in accordance with the local legislation and institutional requirements. Written informed consent for participation was not required for this study in accordance with the national legislation and the institutional requirements.

## Author Contributions

All authors listed have made a substantial, direct, and intellectual contribution to the work, and approved it for publication.

## Funding

This work has been funded by the China postdoctoral foundation grant # 2020M671235.

## Conflict of Interest

The authors declare that the research was conducted in the absence of any commercial or financial relationships that could be construed as a potential conflict of interest.

## Publisher's Note

All claims expressed in this article are solely those of the authors and do not necessarily represent those of their affiliated organizations, or those of the publisher, the editors and the reviewers. Any product that may be evaluated in this article, or claim that may be made by its manufacturer, is not guaranteed or endorsed by the publisher.
